# Case report: A second case of cerebral cavernous malformation after high-dose chemotherapy for medulloblastoma

**DOI:** 10.3389/fonc.2024.1386468

**Published:** 2024-10-30

**Authors:** Maria Grazia Pionelli, Federica Mazio, Maria Elena Errico, Carmela Russo, Adriana Cristofano, Eugenio Maria Covelli, Vittoria Donofrio, Maria Capasso, Michele Antonio Capozza, Fabiola De Gregorio, Serena Ruotolo, Massimo Eraldo Abate, Giuseppe Cinalli

**Affiliations:** ^1^ Pediatric Oncology Unit, Department of Pediatric Onco-Hematology, Santobono-Pausilipon Children’s Hospital, AORN, Naples, Italy; ^2^ Neuroradiology Unit, Department of Neuroscience, Santobono-Pausilipon Children’s Hospital, AORN, Naples, Italy; ^3^ Pathology Unit, Department of Pathology, Santobono-Pausilipon Children’s Hospital, AORN, Naples, Italy; ^4^ Pediatric Neurosurgery Unit, Department of Neurosciences, Santobono-Pausilipon Children’s Hospital, AORN, Naples, Italy

**Keywords:** cerebral cavernous malformation, cavernous hemangioma, cavernoma, medulloblastoma, high-dose chemotherapy

## Abstract

The development of cerebral cavernous malformations (CCMs) is a well-recognized sequela of irradiation to the brain in pediatric tumors, particularly in medulloblastoma, glioma, and acute lymphoblastic leukaemia. So far, only one case of cerebral cavernoma after chemotherapy with autologous hematopoietic stem cell transplantation (HSCT) has been described. We describe a case of a patient with medulloblastoma aged 18 months at the time of oncological diagnosis who was treated with high-dose chemotherapy followed by HSCT and who developed CCM two years later. The patient was not treated for vascular malformation since he remained asymptomatic until now and is regularly followed with neuro-radiological check-ups. This represents the second case of acquired cavernoma developed in a patient who has not received radiation therapy.

## Introduction

Whereas the onset of cerebral cavernous malformations after radiotherapy to the brain in pediatric tumors is a well-known sequela (1) only one case of CCM after high-dose chemotherapy for medulloblastoma has been described (2). Cerebral cavernous malformations (CCMs), also known as cavernous hemangiomas or cavernomas, according to the most recent ISSVA (International Society for the Study of Vascular Anomalies) classification are defined as slow-flow venous malformations, which are vascular abnormalities without endothelial cell proliferation, not tumors (3).

The association between brain irradiation and CCMs is well known. According to Lille’s group (4), radiation-induced cavernomas result from angiogenetic processes and their incidence in patients treated in childhood for brain tumors is 1.3% at 5 years and 7.3% at 20 years.

Less known are the frequency and pathogenesis of CCMs arising after chemotherapy. It has been reported that CCMs developed more frequently and earlier when radiotherapy was associated with chemotherapy (5).

We reported the second case of a patient with medulloblastoma who did not undergo radiotherapy and developed CCM two years after high-dose chemotherapy with HSCT. Before the onset of the cavernoma, our patient already presented two developmental venous anomalies (DVAs). The association between DVAs and cavernomas has been known for some time (6) and the presence of a DVA before the appearance of a therapy-induced cavernoma has already been documented (7).

## Case presentation

**Figure 1:**
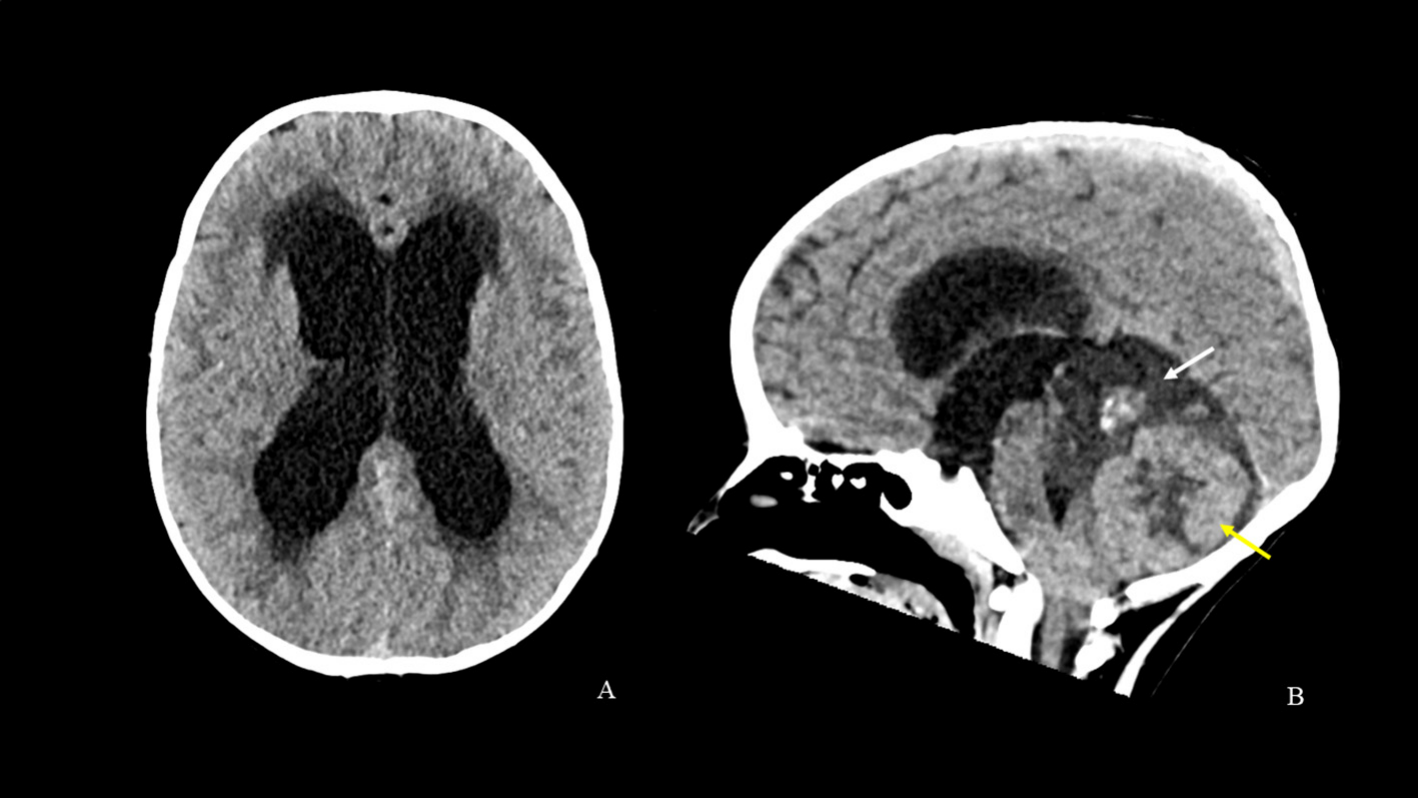
CT scan [axial **(A)** and sagittal **(B)** reconstructions] shows obstructive hydrocephalus and interstitial edema secondary to a posterior cranial fossa tumor. The tumor mass is characterized by a softer hypodense component with a central calcified solid nodule above (white arrow) and by large solid components with central necrotic-cystic phenomena below (yellow arrow). The mass causes compression and displacement of the brainstem and the cerebellar tonsils.

**Figure 2:**
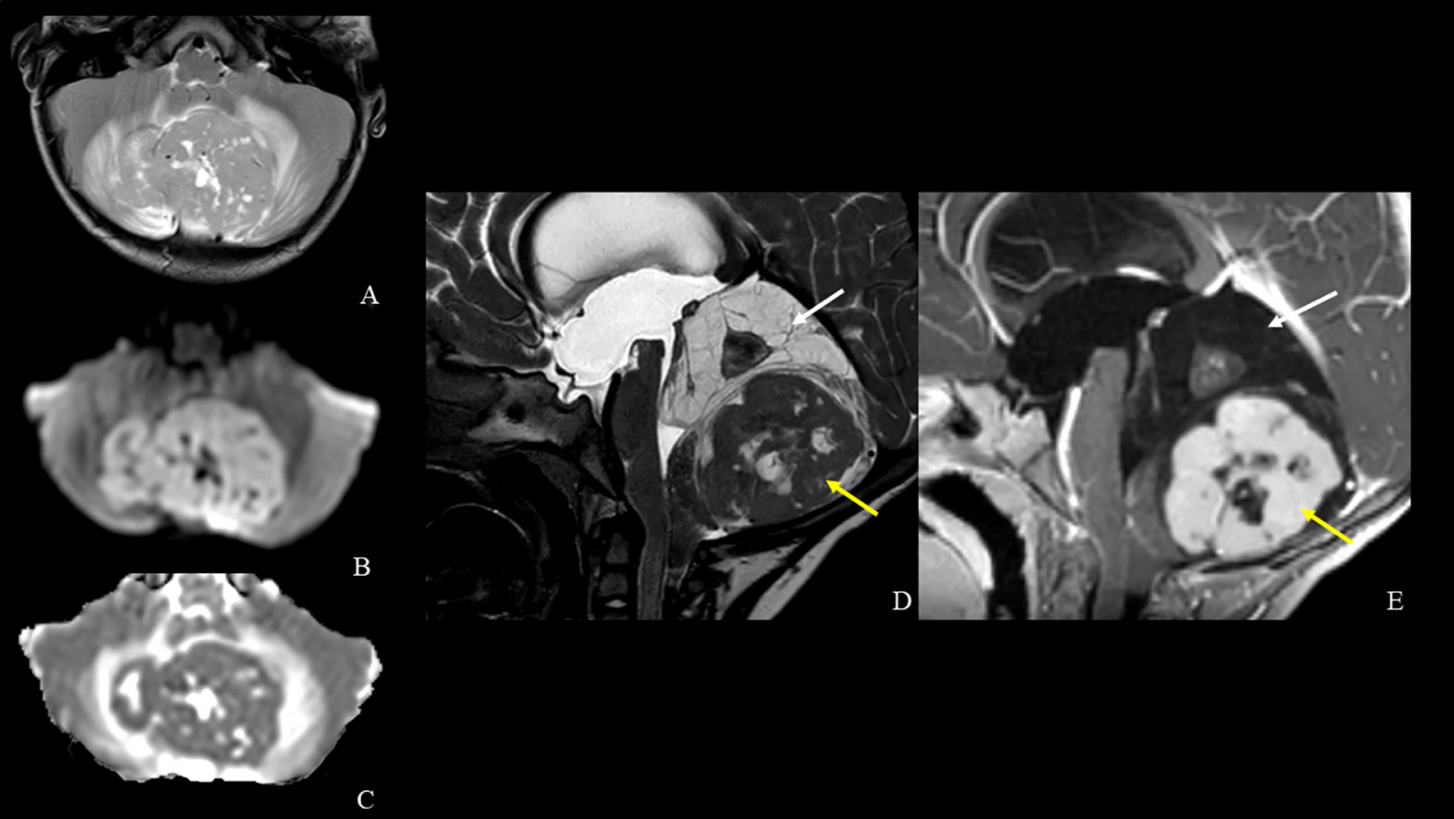
MRI [axial TSE T2-weighted **(A)**, diffusion-weighted imaging **(B)** and ADC map **(C)**, sagittal DRIVE **(D)** and contrast-enhanced T1-weighted **(E)** images] shows a midline vermian tumor with peripheral edema and lateral extension in both cerebellar hemispheres. The tumor mass is heterogeneous and is characterized by a central solid nodule within superior multi-chambered cysts (white arrows) and an inferior larger solid component with low ADC value, necrotic-cystic central core and intense contrast enhancement (yellow arrows).

An 18-month-old boy presented to the emergency room of our hospital with a 10-day history of strabismus and gait disturbances. Neurological examination revealed ataxia and sixth cranial nerve palsy. The remaining physical examination was negative. The past medical history of the patient was silent and he never underwent any interventions. CT scan revealed obstructive hydrocephalus and interstitial edema secondary to the posterior fossa tumor (**Figure 1**). Cranio-spinal MRI showed a midline vermian tumor with peripheral edema and lateral extension in both cerebellar hemispheres. The tumor mass was heterogeneous and characterized by two components. The upper one had a dysplastic-like appearance with multi-chambered cysts and a central solid nodule with calcifications and contrast-enhanced spots. The lower one was composed of large solid confluent nodules with signs of high cellularity (low apparent diffusion coefficient value, ADC value), intense contrast enhancement and necrotic-cystic central core (**Figure 2**). No signs of leptomeningeal dissemination were detected in the brain and spine imaging. Neuro-imaging at initial tumor presentation did not reveal cavernous malformations. His family history was negative for neurological and cardiovascular disease. Since the family history was negative for cavernomas and the patient presented only one lesion, we hypothesized a sporadic form and no genetic investigations were carried out.

**Figure 3:**
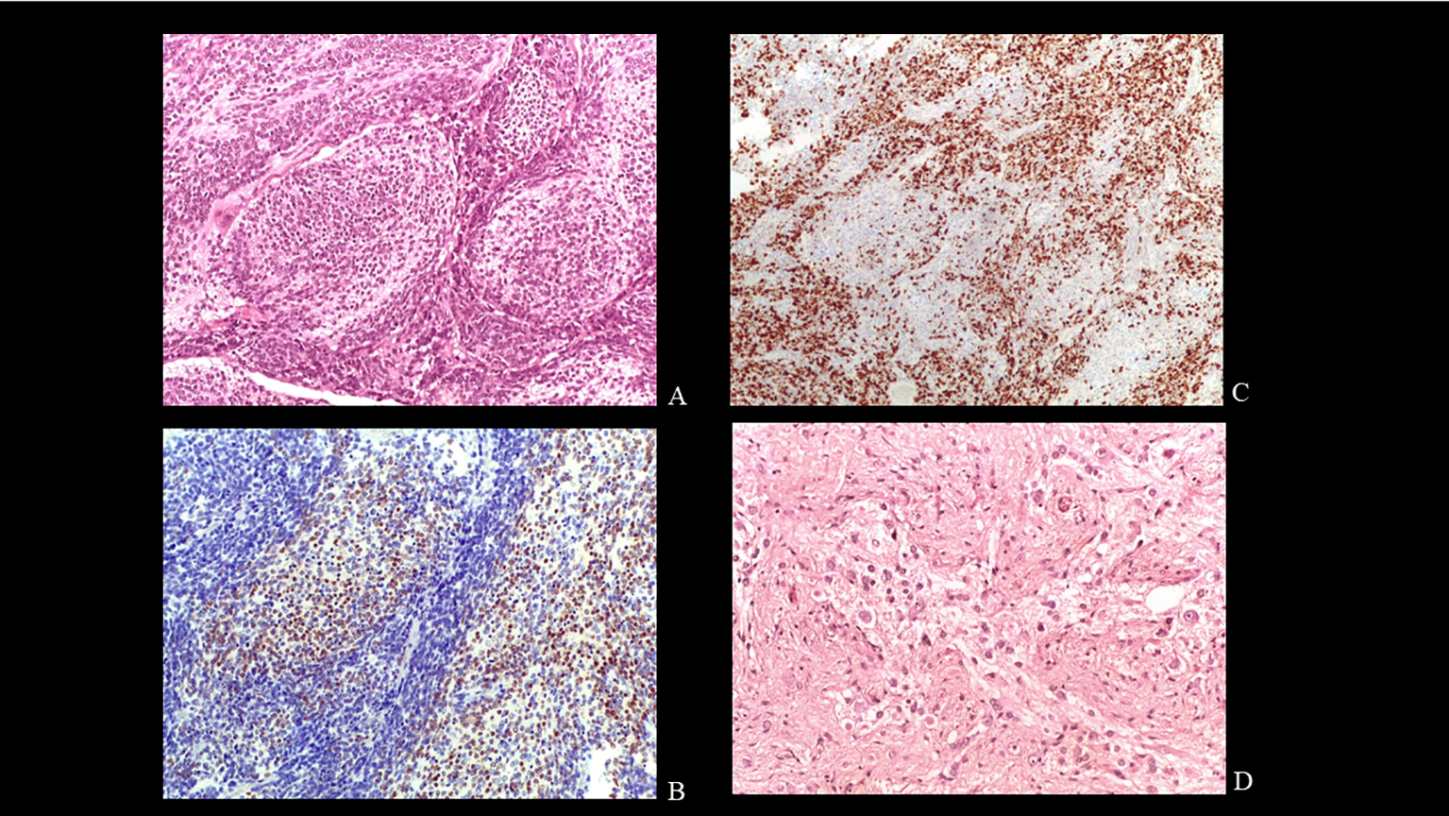
Histology. Pale large nodules composed of uniform small round neurocytic cells in a neuropil-like matrix EE X200 **(A)**. Immunohistochemical reactivity for NeuN in the cells of the pale islands.X200 **(B)**. Strong Ki67 immunolabelling in the internodular regions. X 100 **(C)**. Neurocytic and ganglion cells in post-therapy tumor. EE X400 **(D)**.

He was immediately transferred to the neurosurgery department and underwent resection of the lower portion of the vermian mass without attempting to complete removal. Post-operative MRI performed 24 hours after surgery confirmed the partial removal and absence of complications. The histologic examination revealed an embryonal neoplasm, characterized by large pale nodules of cells with neurocytic differentiation in fine fibrillary matrix, and intervening more cellular, darker areas, composed of poorly differentiated cells, with mitoses and apoptotic debris (**Figure 3A**). Immunohistochemical study showed reactivity of neoplastic cells for synaptophysin and NeuN (in pale islands) (**Figure 3B**) and for GAB, YAP, and FilaminA. Ki67/MIB staining was high (approximately 30%) in the internodular regions (**Figure 3C**). P53 was not expressed. The diagnosis was medulloblastoma with extensive nodularity (MBEN), Sonic hedgehog (SHH) molecular subgroup. Postoperatively, the patient’s clinical course gradually improved and was referred to our department for further treatment. He received four cycles of chemotherapy consisting of methotrexate, vincristine, carboplatin, vepeside and cyclophosphamide. After the second cycle, the patient underwent hematopoietic progenitor cell apheresis (HPC-A) with sufficient harvested stem cells. After a further radiological assessment and a multidisciplinary evaluation, following the third cycle, the patient underwent second-look surgery for residual disease with total removal of the superior portion of the vermian mass. Post-operative MRI confirmed gross total removal. The pathologic examination revealed irregular nodules composed of fibrillary background and clusters of neurocytic and ganglion cells with low proliferative index, consistent with post-chemotherapy maturation (**Figure 3D**). Subsequently, the patient was given two cycles of autologous HSCT with Thiotepa-based conditioning regimen (300 mg/m^2^ for 3 days). The patient was followed with semi-annual clinical and neuro-radiological evaluation.

**Figure 4:**
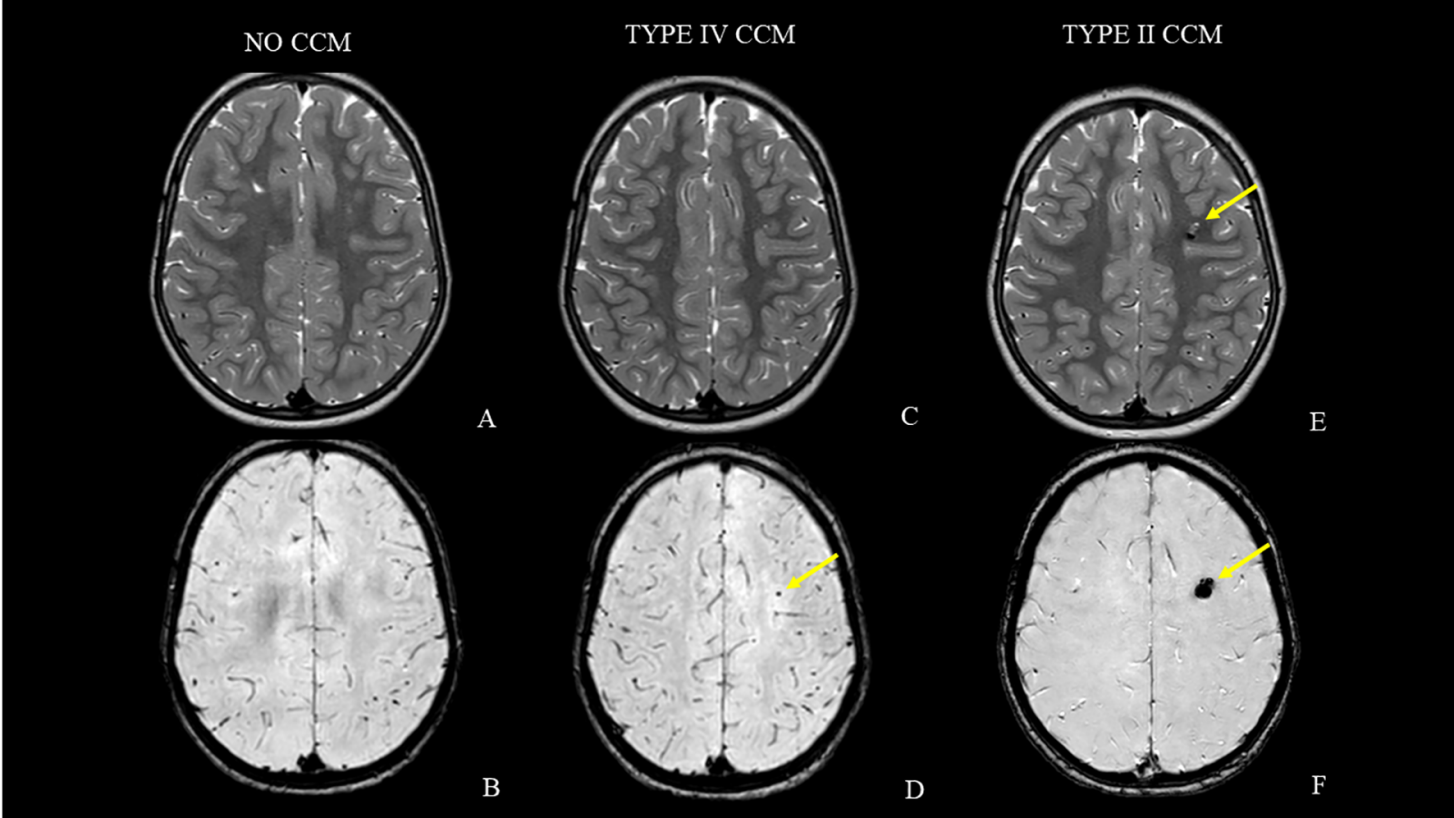
MRI follow-up studies [axial T2-weighted and axial susceptibility-weighted images acquired at case presentation **(A, B)**, after 30 months **(C, D)** and at last MRI follow-up **(E, F)**]. Note the lack of cavernous malformations at the beginning **(A, B)**. The lesion progressively and slowly grew (yellow arrows) and currently has the classic popcorn appearance due to repeated intralesional microbleeds without perilesional edema **(C–F)**.

At 30-month follow-up, the MRI showed a “black dot” with blooming, suspicious for cavernous malformation (type IV according to Zabramski’s classification) in the left frontal white matter in susceptibility weighted imaging (SWI) without signs of recurrence in the posterior fossa. At that time, the patient was neurologically stable, except for mild impaired psychomotor ability and attention and phonological language disorder for which the patient undertook psychomotricity and speech therapy. The CCM progressively and slowly grew in the following two years. It currently has a more heterogeneous structure and has acquired the classic imaging characteristics of cavernoma type II (according to the Zabramski classification) with a popcorn appearance due to repeated intralesional microbleeds. There is no perilesional edema and the patient is still asymptomatic (**Figure 4**). Furthermore, our patient presents two developmental venous anomalies which, although less evident due to hydrocephalus, were already present at the onset of the disease (**Supplementary Figure 1**). The multidisciplinary decision at this time was surgical abstention with regular neuroradiological follow-up.

## Discussion

Cerebral cavernous malformations (CCMs), also known as cavernous hemangiomas or cavernomas, are slow-flow venous malformations without interposed brain parenchyma.

CCMs are angiographically occult. At the microscopic level, the endothelium lacks normal tight junctions, resulting in “leakiness”. Macroscopically, the lesion is likened to a mulberry or raspberry (8). The development of new brain MRI techniques and in particular the routine use of T2*-weighted GRE and SWI sequences in brain protocols has led to an increase in the detection of cavernous malformations. T2*-weighted, SWI, or SWI-like sequences purposely enhance the effect of local field variations caused by tissue content such as blood products (ie, hemosiderin in CCM), iron content (often in the form of ferritin), calcium content, and deoxyhemoglobin in venous blood. These processes cause local variations in the magnetic field that lead to signal loss in the form of T2*. SWI evolved from simple two-dimensional T2*-weighted sequences to three-dimensional sequences with improved spatial resolution and enhanced susceptibility contrast (9). SWI is more sensitive in the diagnosis and detection of the actual number of cavernomas than conventional MRI (10). Bulut et al. showed that SWI is more sensitive than T2*-weighted GRE imaging for detecting CCM in patients with familial cavernomatous disease (11).

Since the onset of the disease, our patient presented two anomalies of venous development. There is known association between DVAs and cavernomas (6) and the presence of a DVA before the appearance of a therapy-induced cavernoma has already been documented. This observation may suggest a predisposition to cavernoma development in this child (7). Our patient’s family history was negative for cavernomas. It has been demonstrated that the rate of association between DVAs and CCMs is higher for sporadic than familial CCMs (12). Evolving data using 7-T SWI suggest that all sporadic CCMs have an associated DVA (13).

CCMs are either sporadic, familial or acquired after irradiation to the brain for pediatric malignancies.

In the sporadic form there is no family history and generally affected individuals have only one lesion. In familial cavernous malformations, it is typical to develop multiple lesions and to have affected family members in consecutive generations. There are 3 known protein-encoding genes resulting in familial CM disease: KRIT1 (CCM1), malcavernin (CCM2), and PDCD10 (CCM3). These genes regulate signaling pathways involved in endothelial tight junction stability, cell proliferation, and angiogenesis (8). The association between brain irradiation and cavernoma formation is well known. The pathogenesis seems to be related to the vascular injury that results in the narrowing of the brain’s smaller blood vessels due to endothelial proliferation with hyalinization and fibrinoid necrosis of the vascular wall (7, 14–17). This leads to ischemia and microinfarction with activation of HIF-1, which in turn induces VEGF causing reactive angiogenesis. Notably, the expression of such growth factor is higher in the very young, potentially explaining the earlier development of CCMs in patients undergoing irradiation at a young age (4, 7, 14, 18). Some studies described a correlation between radiation-induced CCMs and young age (5), but others do not confirm this assumption (1). It has been speculated that the immature brain of the pediatric patient is more sensitive to radiation than the adult brain, and this may contribute to this disproportion of incidence compared to age.

As in some patient subgroups, the incidence of secondary post-RT CCMs is higher and the time to their manifestation is shorter than in other patients, genetic or epigenetic heterogeneity may be involved (14). On the other hand, the literature surrounding the impact of chemotherapy alone in the development of CCMs is lacking. Our case is the second one reported to date describing a patient treated with high-dose chemotherapy for medulloblastoma who developed a secondary CCM without receiving radiation therapy.

Medulloblastoma is the most common primary tumor associated with acquired CCM formation, as reported in three large case series (1, 5, 19). Advances in characterizing histological subtypes of medulloblastoma have enabled molecular patient stratification for treatment decision-making (20). More recent strategies to delay or avoid craniospinal radiotherapy, especially in younger children, have provided evidence for improved survival rates by intensive systemic and intraventricular chemotherapy alone or by intensified systemic chemotherapy and high-dose, marrow-ablative chemotherapy with or without radiotherapy (21). In young children with desmoplastic/nodular (DMB) and extensive nodular (MBEN) subtype of medulloblastoma, postsurgical treatment is based on chemotherapy alone (21–23).

MBEN is rare and occurs mainly in infants, accounting for less than 3% of medulloblastomas (24). The tumor reveals signs of neuronal differentiation and, following radiotherapy and/or chemotherapy, may occasionally undergo further maturation to tumors predominantly composed of ganglion cells (25).

Some authors speculated on the possible added risk of chemotherapy in cavernoma formation (7, 14). Di Giannatale et al. reported 27 cases of children treated for medulloblastoma who developed cavernomas. CCMs developed mainly in patients who received methotrexate plus radiotherapy than those who received only radiotherapy (70% versus 30%), and the latency was shorter in the same group of patients (50% in the first 6 years for methotrexate followed by radiotherapy versus 11 years for radiotherapy only) (5). The potential additional risk of methotrexate is supported by the documentation of small-vessel disease in children with acute lymphoblastic leukemia (ALL) previously treated with intravenous and intrathecal methotrexate (5). ALL is the third most common malignancy associated with radiation-induced cavernous malformations (1, 26, 27). It is possible that in patients with ALL, both cranio-spinal irradiation and the use of such chemotherapy are responsible for the development of intracranial cavernous malformations.

However, no significant relationship between cavernoma development and chemotherapy drugs has been clearly described to date (14, 17). Yamasaki et al. postulated that vascular damage including damage of capillaries by anti-angiogenic cytotoxic chemotherapeutic agents in patients who underwent high-dose chemotherapy may be involved in the development of secondary CCMs. Vascular toxicity is a frequent adverse effect of current anticancer chemotherapies resulting from endothelial dysfunction. Chemotherapy can provoke direct endothelial damage, activation of coagulation factors, autonomic neuropathy, dysfunction of smooth muscle cells in the terminal arterioles, vasculitis and augmentation of fibroblast proliferation (14). On the other hand, chemotherapy-induced cognitive impairment is a common adverse effect of cytotoxic chemotherapeutic drugs. Younger age at treatment and higher intensity therapy schemes have been associated with worse neurocognitive outcomes in brain tumors of childhood, most likely resulting from the greater vulnerability of the developing brain to neurotoxic agents, with or without irradiation (28).

Although cavernous malformations are often incidental findings, the sequelae associated with their bleeding can be severe, including focal neurologic deficits and seizures, with patients requiring close follow-up and possible surgical intervention (1, 29).

## Conclusions

Existing literature about the possible development of secondary CCM in patients treated with high-dose chemotherapy for pediatric tumors is scarce with only another case described until now. We presented the second case of a patient affected by medulloblastoma treated with high-dose chemotherapy followed by HSCT who developed CCM during its follow-up.

Knowledge of potential secondary changes in the brain such as CCMs in patients undergoing high-dose chemotherapy makes regular long-term follow-up highly recommended to identify them earlier and avoid complications. Further studies are needed to better understand the role of chemotherapy in the onset of CCMs, their incidence and the possible predictive value of a pre-existing developmental venous anomaly.

## Data Availability

The raw data supporting the conclusions of this article will be made available by the authors, without undue reservation.

## Supplementary material

Supplementary Figure 1 MRI studies [axial and coronal contrast-enhanced T1-weighted images acquired at disease presentation **(A, B)** and last MRI follow-up **(C, D)**]. Note the presence of two DVAs, located in the left frontal antero-medial area (white arrows) and in the right cingulum (yellow arrows).
