# Supplementary figures and images for: Case report: A second case of cerebral cavernous malformation after high-dose chemotherapy for medulloblastoma
Source: Front Oncol. 2024 Oct 30;14:1386468. doi: 10.3389/fonc.2024.1386468 (PMC11557518; doi:10.3389/fonc.2024.1386468)

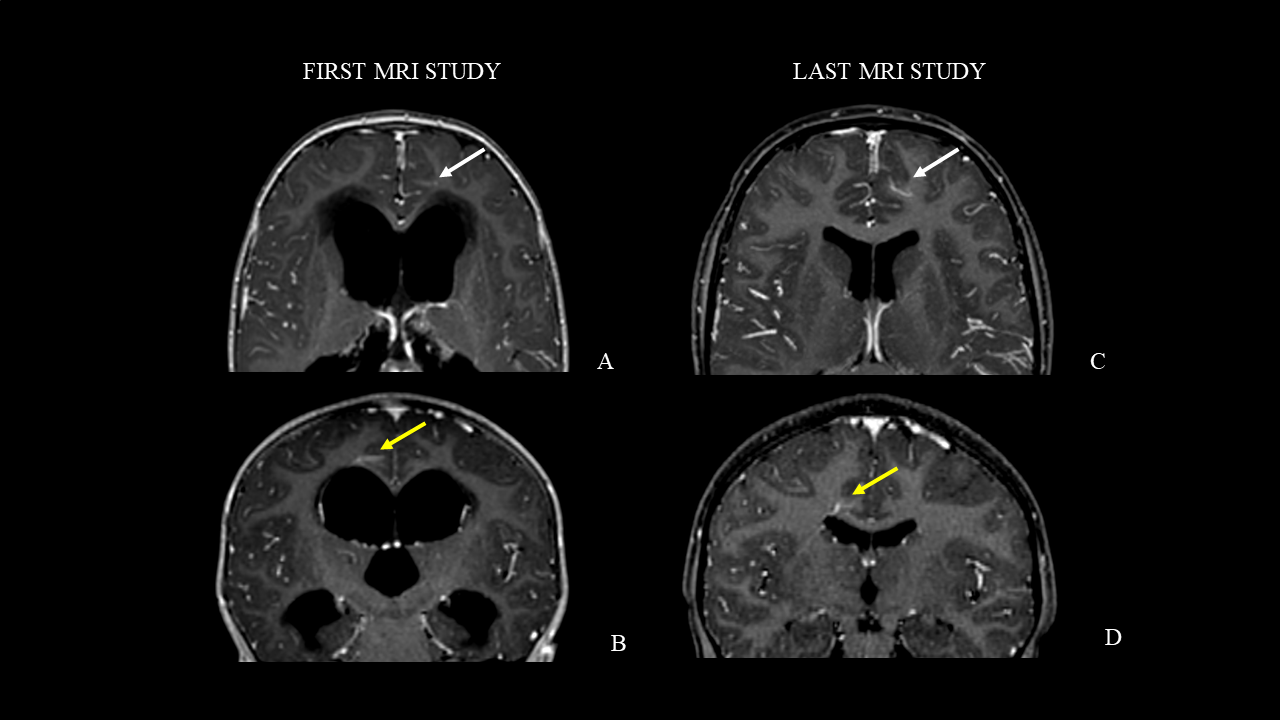

Supplement: Supplementary Figure 1 — MRI studies [axial and coronal contrast-enhanced T1-weighted images acquired at disease presentation (A, B) and last MRI follow-up (C, D)]. Note the presence of two DVAs, located in the left frontal antero-medial area (white arrows) and in the right cingulum (yellow arrows). [file Image1.tif]
